# The feasibility of an allergy management support system (AMSS) for IgE-mediated allergy in primary care

**DOI:** 10.1186/s13601-018-0206-y

**Published:** 2018-05-29

**Authors:** Bertine M. J. Flokstra-de Blok, Thecla M. Brakel, Marian Wubs, Ben Skidmore, Janwillem W. H. Kocks, Joanne N. G. Oude Elberink, Marie-Louise A. Schuttelaar, Jantina L. van der Velde, Thys van der Molen, Anthony E. J. Dubois

**Affiliations:** 1 University of Groningen, University Medical Center Groningen, Department of General Practice, Internal Postcode FA21, PO Box 196, 9700 AD Groningen, The Netherlands; 2 University of Groningen, University Medical Center Groningen, GRIAC Research Institute, Groningen, The Netherlands; 3 University of Groningen, Teaching Unit, Department of Social Psychology, Groningen, The Netherlands; 4 University of Groningen, University Medical Center Groningen, Department of Allergology, Groningen, The Netherlands; 5 University of Groningen, University Medical Center Groningen, Department of Dermatology, Groningen, The Netherlands; 6 University of Groningen, University Medical Center Groningen, Department of Pediatric Pulmonology and Pediatric Allergy, Groningen, The Netherlands

**Keywords:** Allergy, Diagnosis, Feasibility, Management support system, Primary care

## Abstract

**Background:**

The allergy management support system (AMSS) was developed to assist general practitioners (GPs) to handle the increasing burden of allergic diseases and facilitates the diagnosis and management of allergy. The aim of this cluster-randomized controlled pilot study was to test the feasibility of this AMSS for primary care.

**Methods:**

GPs received diagnostic and management recommendations generated by the AMSS in addition to sIgE-test results (intervention) or GPs received sIgE-test results only (control). The AMSS recommendations are based on the previously developed patient-completed AMSS questionnaire and sIgE-test results. The AMSS was considered feasible when > 70% of the AMSS recommendations were sent to the GP within ten working days of sIgE-testing. GPs completed a questionnaire on their diagnosis and management before (T1) and after (T2) receiving sIgE test results. Agreement and disagreement concerning diagnosis, medication and referrals between GPs and AMSS was investigated at T1 and T2. A total agreement score between GPs and AMSS was calculated. GPs in the intervention group completed a questionnaire to evaluate the utility of the AMSS. Semi-structured interviews were used to explore the motivation of GPs who did not include patients in this pilot study.

**Results:**

Twenty-seven GPs included 101 patients. Forty-two patients (72%) completed the AMSS questionnaire in the intervention group. The majority of the AMSS recommendations (93%) were returned to the GP within 10 working days after sIgE-test results were known [mean (SD) 4.7 (4.0) working days]. GPs in the intervention group reported largely following the AMSS recommendations in 71% of cases. The total agreement scores concerning diagnosis were significantly higher (p < 0.001) in the intervention group than the control group [mean (SD); 0.9 (1.8) vs − 0.8 (1.0)]. The agreement concerning medication or referral between GPs and AMSS did not differ between the intervention and the control group. GPs in the intervention group were reasonably positive about the AMSS. Not enrolling patients was not caused by anticipated ineffectiveness of the AMSS.

**Conclusion:**

The AMSS can be considered to be feasible for primary care. GPs tend to follow the AMSS recommendations. The AMSS may contribute to the empowerment of GPs to better manage allergy patients in primary care.

*Trial registration* ISRCTN ISRCTN36780877. Registered 23 November 2017 (retrospectively registered)

## Background

Allergy is one of the most common chronic diseases with the number of people suffering from allergic diseases increasing worldwide [1–4]. Currently, more than 150 million people in Europe experience allergic symptoms [5]. The number of certified allergists per head of population ranges from 1:1,240,000 to 1:16,000 in European countries [6]. About 50% of all children under 18 years visit their general practitioner (GP) for allergic symptoms [7]. Diagnosis and management of patients suspected of allergy is often performed by GPs. In many cases, especially where there are very few allergists operating in large populations, GPs are exclusively responsible for providing allergy care [8].

GPs report having difficulties in diagnosing and managing allergic patients, including the interpretation of specific IgE (sIgE), which they attribute to a lack of allergy training [9]. Of the major allergic diseases, food allergy has been reported by GPs as the most troublesome [10, 11]. In addition, it has been shown that of the case mix of patients referred to a regional allergy service by GPs, only 43% of patients were diagnosed with an IgE mediated allergic disease [12]. In the United Kingdom, it has been reported that probably a quarter and maybe half of allergy referrals to secondary care may be dealt with in primary care by a GP with special interest in allergy [13]. These results demonstrate that allergy management in primary care is not optimal in most European countries [14].

Since the specialist-based allergy care model will become unsustainable with increasing allergy prevalence, a new allergy care model with a more important role for the GP is mandatory [8]. Therefore, we developed an allergy management support system (AMSS) to support GPs in diagnosing and managing patients with IgE-mediated allergy in primary care [11, 15]. Based on a patient-reported history questionnaire and specific IgE (sIgE) blood test results, we could demonstrate that the AMSS is able to provide a probable diagnosis and recommendations on management of allergic disorders for GPs [15]. The primary aim of this pilot study was to test the feasibility of an AMSS for primary care. In addition, preliminary effects of the AMSS on diagnosis, medication and referrals were investigated. Finally, an evaluation of the AMSS by the GPs was carried out.

## Methods

### Study design

Using a cluster randomized controlled design, GPs in the intervention group received AMSS recommendations in addition to sIgE test results, while GPs in the control group performed usual care based on sIgE test results only. GPs included patients in the study during a 6-month period (from January 2014 until July 2014). This pilot study was approved by the local medical ethics committee (METc 2013/129) who deemed that the study did not fall within the Dutch Medical Research Involving Human Subjects Act. Participation was voluntary, all participants received written information about the study and all participants signed an informed consent form.

### Participants

All GPs allied to a single regional GP laboratory in the northern part of the Netherlands were invited to participate in this pilot study. The practices of GPs that agreed to participate were randomized to the intervention or the control group. The GPs included eligible patients (both children and adults). The only inclusion criterion was that the GP had ordered a sIgE test (usually screening of inhalant allergens (grass pollen, tree pollen, house dust mite, cat, dog, moulds, weed pollen) or food allergens (hen’s egg, milk, cod, wheat, peanut, soy) for that particular patient regarding an allergy related problem. There were no explicit exclusion criteria. A formal power calculation was not applicable, since this is a pilot study which aims to investigate the feasibility of the AMSS for primary care.

### The allergy management support system (AMSS)

**Fig. 1:**
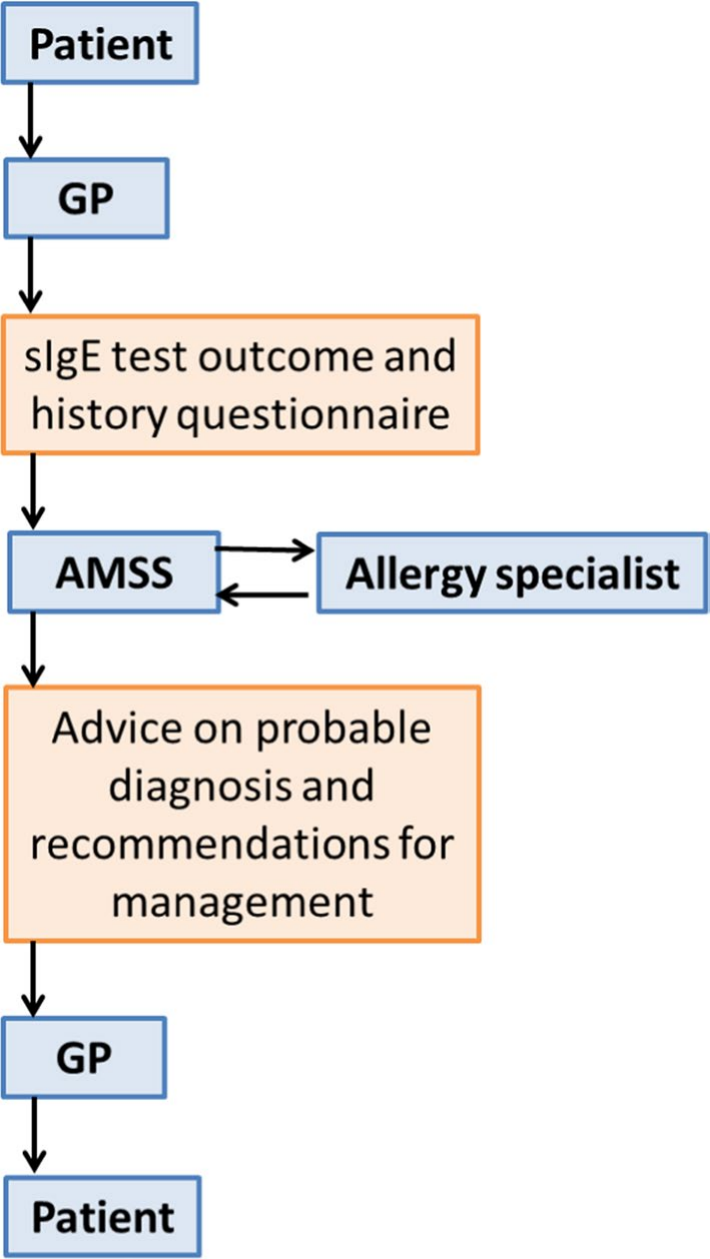
Schematic representation of the allergy management support system (AMSS) [15]

Participating patients in the intervention and control group completed the AMSS questionnaire and sent the questionnaire to the AMSS researchers for analysis together with the sIgE test results. Previously developed algorithms (for adults and children) were used to allocate patients to predefined diagnostic categories and consequent recommendations for management [15]. In addition, the AMSS questionnaire, sIgE test results and resultant AMSS recommendations were checked by allergy specialists for inaccuracies or ambiguities. An AMSS advice was formulated for patients in the intervention and control group. The AMSS recommendations, together with a copy of the completed AMSS questionnaire and the sIgE test outcome, were sent only to the GPs in the intervention group by post (Fig. 1). The AMSS recommendations formulated for patients in the control group were for evaluative purposes only and were not made available to the GPs in the control group.

### Measures in patients

Participating patients in the intervention and control group completed the AMSS questionnaire which consists of 12 (mainly) multiple-choice questions on symptoms, triggers, severity, medication and symptom control of their allergic condition(s) [15].

### Measures in GPs

#### Efficacy measure and case-specific evaluation

GPs in both groups completed a short questionnaire with multiple-choice questions on diagnosis and management (medication, referral and/or non-pharmacotherapeutic recommendations). This questionnaire was completed on two occasions: at the time of inclusion of the patient (T1) and at the time the sIgE test outcomes (and in the case of the intervention group, the AMSS recommendations) were known (T2). In addition, the T2 questionnaire included a question on the GPs’ satisfaction with their own management of the patient (5-point scale ranging from very unsatisfied to very satisfied). For GPs in de intervention group the T2 questionnaire also included three multiple-choice questions to evaluate the case-specific AMSS recommendations.

#### Process evaluation

At the end of the inclusion period, the GPs in the intervention group completed a questionnaire to evaluate the usefulness of all the AMSS recommendations they received during the study and to evaluate the utility of the AMSS for primary care. This questionnaire consisted of 13 multiple-choice questions and one question to rate the AMSS system with a score between 1 (worst) and 10 (best).

#### Qualitative evaluation

After the inclusion period, it turned out that 27 (36%) of the 75 GPs included patients in the study. Qualitative research was undertaken to eliminate possible (self)selection bias and ascertain the generalizability of the results. Semi-structured interviews were used to explore the motivation of GPs who did not include patients in the pilot study. In order to obtain diversity in the qualitative sample, GPs were purposively selected based on the allocation of the GP to the intervention or control group, sex of the GP and location of the GP’s practice. GPs were interviewed until saturation was reached. Interviews were transcribed verbatim and analyzed using Atlas.ti based on the grounded theory [16, 17].

### Statistical analysis

The AMSS was considered feasible if > 70% of the AMSS recommendations was sent to the GP within 10 working days of sIgE testing. Descriptive statistics included mean (SD) and percentages. Non-parametric statistics were performed using Mann–Whitney U test or Fisher exact test. Agreement and disagreement concerning diagnosis, medication and referrals between GPs and AMSS were investigated at T1 and T2. For each patient, agreement was the number of diagnoses made by both the AMSS and the GP. There were two types of disagreement: the first was the number of diagnoses made by the AMSS but not by the GP (referred to hereafter as disagreement A) and the second was the number of diagnoses made by the GP but not by the AMSS (disagreement B). A total agreement score was then calculated as agreement minus both disagreement A and B. Finally, the change of the scores were calculated as T2 scores minus T1 scores. The same methodology was used for (dis)agreement concerning medication prescriptions and referrals. The data was analysed using SPSS22.0 (IBM, Chicago, USA).

## Results

**Fig. 2:**
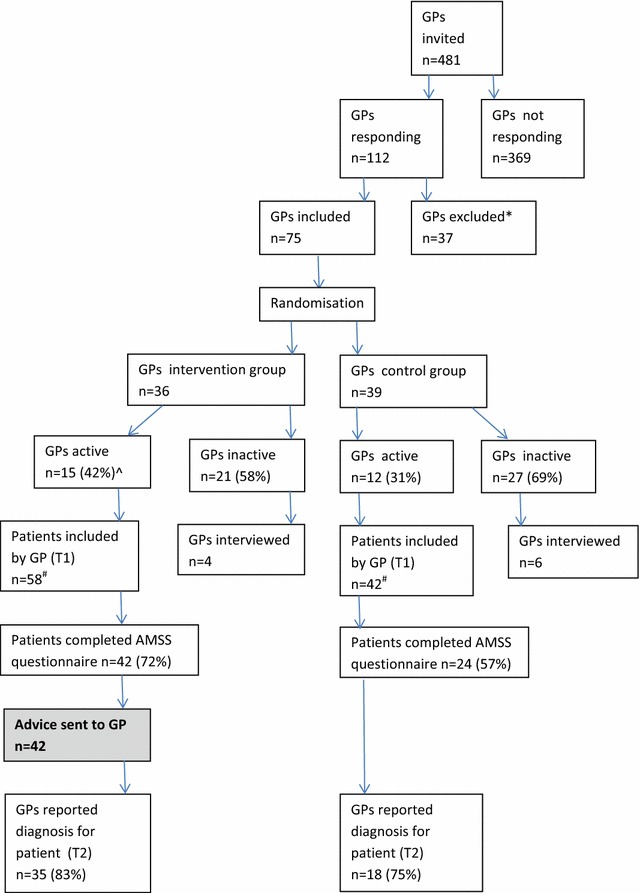
Flow chart of the pilot study. *GP refused to participate because of being busy/no priority (n = 17), did not expect relevant cases (n = 4), moving/changes in practice (n = 6), performs sIgE at other laboratory (n = 4), responded too late (n = 2) or other reason (n = 4). ^Percentages represent those of the previous step in the chart. ^#^In total 101 patients were included by GPs. However, for one patient it was unclear by which GP she was included and thus whether the GP was from the intervention or control group. Also, she did not complete the AMSS questionnaire and was therefore excluded from further analysis

In total 481 GPs were invited to participate in this pilot study, of which 75 GPs agreed to participate and 27 GPs included at least one patient (37% active GPs). Together they included 101 patients of which 66 (66.7%) completed the AMSS questionnaire (Fig. 2). Table 1 describes the included GPs and patients. There was no significant difference in the number of (preliminary) diagnoses reported by the GPs in the intervention versus the control group.

**Table 1:** Descriptive characteristics of the GPs and the patient characteristics at T1

	Intervention group (n = 36)	Control group (n = 39)
*GPs*		
Sex, n (m/f)	25/11	28/11
Households in community, n (< 20,000/> 20,000)	23/13	27/12

	Intervention group (n = 58)	Control group (n = 42)
*Patients*		
Sex, n (m/f)	18/39^b^	13/29
Age, mean in years (SD)	28.7 (18.6)	28.5 (17.9)
Age, n (child/adult)	20/38	16/26
Diagnosis by GP, n (%)^a^		
Allergic rhinitis	36 (62)	24 (57)
Asthma	11 (19)	10 (24)
Eczema	5 (9)	4 (10)
Bee/wasp allergy	0	0
Medication allergy	0	2 (5)
Latex allergy	0	0
Food allergy	9 (16)	8 (19)
No preliminary diagnosis	3 (5)	3 (7)
Other	12 (21)	6 (14)
Medication prescribed by GP, n (%)	33 (57)	28 (67)
Other management recommendations by GP, n (%)	29 (50)	21 (50)
Referred by GP, n (%)	3 (5)	3 (7)

^a^More than one category was possible

^b^One missing

### Feasibility

The majority of the AMSS recommendations (93%) were sent back to the GP within 10 working days after the sIgE test results were known [mean (SD) 4.7 (4.0) days].

### Case-specific evaluation of AMSS recommendations

At T2, GPs in the intervention group reported that the AMSS recommendations were complete and to-the-point in 80% of cases, that they largely agreed with the AMSS recommendations in 80% of cases and that they largely (71% of cases) or partly (20% of cases) followed the AMSS recommendations. Specific patterns for not (or partly) following the AMSS recommendations could not be determined.

### GPs’ satisfaction with their own management

At T2, GPs in the intervention group reported being satisfied to very satisfied regarding the diagnosis and management recommendation for their patient in 88% of cases, whereas GPs in the control group reported this in 72% of cases (n.s.).

### Agreement between AMSS and GPs concerning diagnosis

**Fig. 3:**
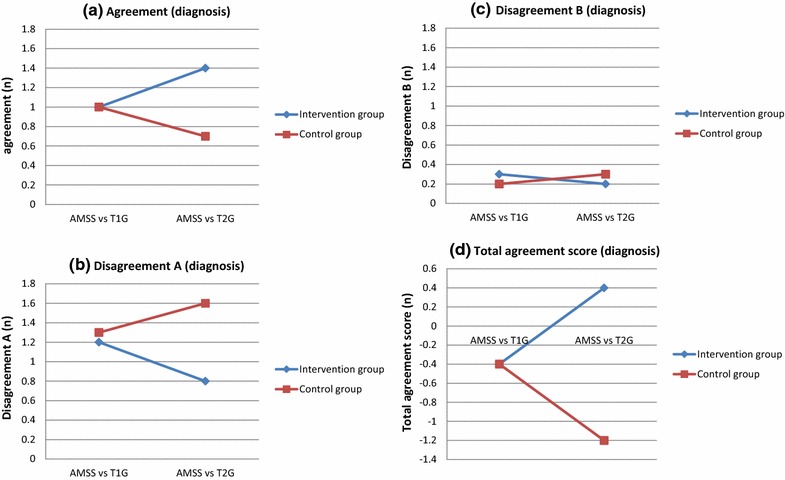
Agreement concerning diagnosis by AMSS and diagnosis reported by GPs (T1 and T2). Selection based on complete data sets at T1 and T2 (intervention group n = 35, control group n = 18). **a** Agreement (GP +/AMSS +), **b** disagreement A (AMSS +/GP −), **c** disagreement B (AMSS −/GP +), **d** total agreement score (agreement minus both disagreement A and B)

At T2, significantly more agreement (AMSS +/GP +) (p = 0.016), less disagreement A (AMSS +/GP −) (p = 0.004) and a higher total agreement score (agreement minus both disagreement A and B) (p = 0.003) concerning diagnosis were found for the intervention group compared to the control group. Also, for the change of scores (T2 minus T1), significantly more agreement (p = 0.001), less disagreement A (p < 0.001) and a higher total agreement score (p < 0.001) were found for the intervention than for the control group. Disagreement B did not differ significantly between the groups (Fig. 3a–d). Table 2 shows the diagnoses reported by the GPs in the intervention group and control group at T1 and T2 and the probable diagnosis by the AMSS.

**Table 2:** Diagnoses^a^ reported by the GPs in intervention group and control group at T1 and T2 and the probable diagnosis by the AMSS (more than one diagnosis possible per patient)

	Intervention group (n = 35)			Control group (n = 18)		
	T1	AMSS	T2	T1	AMSS^b^	T2
Allergic rhinitis	20 (57)	33 (94)	22 (63)	10 (56)	17 (94)	7 (39)
Asthma	9 (26)	25 (71)	14 (40)	7 (39)	15 (83)	6 (33)
Eczema	4 (11)	4 (11)	5 (14)	1 (6)	4 (22)	1 (6)
Bee/wasp allergy	0	2 (6)	0	0	0	0
Medication allergy	0	1 (3)	1 (3)	0	1 (6)	0
Latex allergy	0	1 (3)	0	0	0	0
Food allergy	5 (14)	8 (23)	6 (17)	3 (17)	0	2 (11)
Urticaria	1 (3)	1 (3)	1 (3)	0	0	0
No diagnosis	3 (9)	0	1 (3)	0	1 (6)	3 (17)
Other	8 (23)	2 (6)	7 (20)	2 (11)	5 (28)	3 (17)

^a^Presented as n (%)

^b^GPs in the control group did not receive probable diagnoses from the AMSS. The AMSS formulated these probable diagnoses for evaluative purposes only

### Agreement between AMSS and GPs concerning medication

Table 3 shows the medication reported by the GPs in the intervention group and control group at T1 and T2 and the medication recommendations of the AMSS. No significant differences between intervention and control group were found regarding agreement and disagreement between AMSS and GPs concerning medication, except disagreement A (AMSS +/GP −) at T2 (p = 0.026) with less disagreement in the intervention group (data not shown).

**Table 3:** Medication^a^ reported by the GPs in intervention group and control group at T1 and T2 and the medication recommendations of the AMSS

	Intervention group (n = 35)			Control group (n = 18)		
	T1	AMSS	T2	T1	AMSS^b^	T2
Antihistamines						
Nasal	2 (6)	1 (3)	3 (9)	0	1 (6)	0
Ocular	2 (6)	0	2 (6)	1 (6)	0	1 (6)
Oral	10 (29)	20^c^ (57)	15 (43)	4 (22)	7 (39)	4 (22)
Corticosteroids						
Nasal	9 (26)	16 (46)	15 (43)	3 (17)	9 (50)	4 (22)
Pulmonary	4 (11)	5 (14)	6 (17)	2 (11)	2 (11)	1 (6)
Oral	0	0	0	1 (6)	0	1 (6)
Cutaneous class 1	1 (3)	2 (6)	1 (3)	0	2 (11)	0
Cutaneous class 2	1 (3)	3 (9)	1 (3)	0	3 (17)	0
Cutaneous class 3	0	0	0	1 (6)	1 (6)	1 (6)
Cutaneous class 4	0	0	0	0	0	0
B-sympathicomimetics						
Short-acting	7 (20)	20 (57)	9 (26)	3 (17)	13 (72)	4 (22)
Long-acting	0	0	1 (3)	0	1 (6)	0
Leukotrien antagonists	0	0	0	1 (6)	0	1 (6)
Combination therapy (corticosteroids with B-sympathicomimetics)	0	1 (3)	1 (3)	2 (11)	0	2 (11)
Decongestants	0	0	0	0	0	0
Emollients	1 (3)	7 (20)	1 (3)	1 (6)	5 (28)	1 (6)
Epinephrine auto-injector	0	8 (23)	2 (6)	0	3 (17)	0

GPs did not report considering immunotherapy for any patients. The AMSS advised immunotherapy as a follow-up option after instituting pharmacotherapy 16 times (46%) in the intervention group and 9 times (50%) in the control group. The AMSS advised venom immunotherapy for a single patient

^a^Presented as n (%)

^b^GPs in the control group did not receive AMSS recommendations. The AMSS formulated these recommendations for evaluative purposes only

^c^For two persons the AMSS advised H2 antagonist as urticaria medication

### Agreement between AMSS and GPs concerning referrals

At T1, GPs in the intervention and control group reported 3 (9%) and 1 (6%) referrals, respectively. The AMSS advised 9 (26%) and 5 (29%) referrals in the intervention and control groups, respectively. At T2 the GPs in the intervention and control group reported 10 (29%) and 3 (18%) referrals, respectively (p = 0.504). No significant differences between intervention and control group were found regarding agreement and disagreement between AMSS and GPs concerning referrals (data not shown).

### Process evaluation

**Fig. 4:**
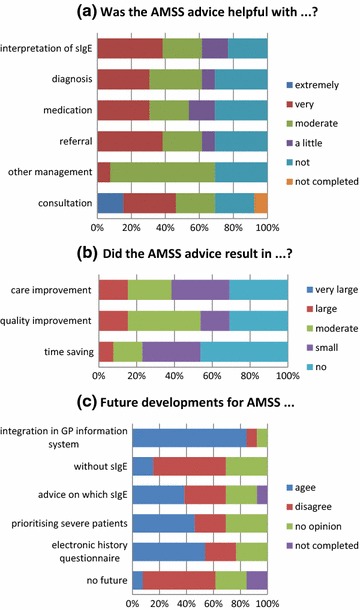
**a** Helpfulness of the AMSS recommendations, **b** improvements brought about by the AMSS, **c** further developments of the AMSS (as indicated by the GPs in the intervention group)

Of the 15 active GPs in the intervention group, 14 returned the overall evaluation questionnaire. However, one questionnaire was returned blank, thus 13 questionnaires were eligible for analysis. The AMSS was rated by the GPs with an average score of 6.7 out of 10. The majority found the AMSS recommendations helpful in making the diagnosis and determining the management of patients (Fig. 4a). A majority also found that the AMSS led to (at least a small) improvement in care and quality (Fig. 4b) and wished the AMSS to be integrated into the GP information system in the future (Fig. 4c).

### Qualitative evaluation

Saturation occurred after ten interviews with inactive GPs (i.e. randomized GPs who did not include patients). GPs mentioned several reasons for not including patients into this study: lack of time (n = 6), not appreciating the importance of the study (n = 5), and forgetting about the study (n = 4). All GPs emphasized the importance of reminders in order to stay involved. Younger GPs preferred an email as reminder and older GPs preferred a visit. No GP indicated that the AMSS system was anticipated to be ineffective, or unworkable.

## Discussion

The results of this pilot study suggest that the AMSS is feasible in primary care. In addition, a majority of the GPs reported that they completely followed the AMSS recommendations. This was confirmed by the greater agreement concerning diagnoses between GPs and AMSS in the intervention group compared to the control group (usual care). The agreement concerning medication or referral between GPs and AMSS did not differ between the intervention and the control group. The active GPs in the intervention group were reasonably positive about the AMSS and foresaw the usefulness of the AMSS in the future.

To our knowledge this is the first study that investigates a comprehensive allergy support tool in real primary care practices. In the past, only one partly comparable initiative was undertaken in the allergy field [18]. However, that system added simple standardized sentences to sIgE test outcomes and was not based on an extensive, structured clinical history. The various reasons mentioned by inactive GPs who did not enrol patients in our current pilot study do not suggest that non-participation was due to disapproval or anticipated disappointment with the AMSS. This is an important finding in the light of computer decision support systems within routine care. At least in asthma it has been shown that these support systems are rarely used and the advice is not followed [19]. This is in contrast to our study where we demonstrated that the GPs were following the AMSS advice.

Initially, we hypothesized that the AMSS would prevent referrals. However, in this study GPs in the intervention group tend to refer patients to specialist care more often than GPs in the control group. It may be that GPs in the control group performing usual care underestimated the problems and severity of allergic patients, whereas the GPs in the intervention group were alerted by AMSS recommendations which encouraged them to refer the patient on to specialist care. In principal, this may not be a bad thing, as for example one would not wish to delay the referral of a patient with sIgE mediated anaphylaxis [20]. Based on these findings, the emphasis for future work may shift from providing GPs with recommendations allowing them to manage more allergy patients in primary care, to identifying in which environment (primary or specialist care) the care of the allergic patient is likely to be optimized. This suggests perhaps that the AMSS, after further refinements may also be used as a tool to enable care stratification.

A potential strength of the AMSS in the future is the possible learning effect for GPs using this system. By receiving individualized patient-specific feedback on each case, GPs could become more effective in identifying, managing and referring allergic patients. This will also be expected to have a learning effect on whether sIgE determinations are required or not and how they should be interpreted. In this way, the AMSS meets the unmet need of GPs having difficulty with interpretation of sIgE test results [9, 21]. Moreover, this direct patient-related learning is likely to improve care for patients with allergies in primary care [22, 23]. In addition, the AMSS should be computerized in the future and recommendations should preferably be accessed via the GP’s information system in a digital format [19]. Other future developments may include using the AMSS to guide and rationalize sIgE testing and prioritizing referral to secondary care based on severity as ascertained by the AMSS. These developments could further improve the efficiency of the AMSS and may contribute to the development of an integrated care pathway for allergic patients [24, 25].

One of the strengths of this study is that it was performed in real primary care practices using a cluster randomized design. Therefore it gives robust information on practical issues relating to the use of the AMSS, such as performance and feasibility. In addition, it also provides preliminary effect measurements. Furthermore, the randomized pilot study was enriched with a questionnaire-based evaluation under the active GPs in the intervention group and a qualitative evaluation under the inactive GPs, together providing a broad insight into the potential utility of the AMSS in primary care.

A limitation of this study is that the AMSS questionnaire is not extensively validated. However, in an earlier study we showed good agreement (69.2%) between specialist’s recommendations (gold standard) and recommendations generated by the AMSS [15]. In addition, this pilot study showed that the AMSS questionnaire is feasible to advise GPs on diagnosis and management of allergic patients in primary care and in that sense the questionnaire showed face validity. In addition, the generalizability of the study may be questioned because of the relatively low number of included patients. However, since this was a pilot study we had neither the intention nor the capacity to include large numbers of patients. Larger numbers of patients are needed to further validate, refine and computerize the AMSS before rolling it out to full scale. Finally, it should be noted that a history questionnaire such as used in the AMSS is not intended to replace a specialist consultation, but rather to support GPs. GPs will remain ultimately responsible and autonomous and may always decide to deviate from the recommendations of the AMSS.

## Conclusion

In conclusion, the AMSS may be considered feasible for primary care. A majority of the GPs reported that they followed the AMSS recommendations completely, that they were reasonably positive about the utility of the AMSS, and that they were optimistic about future developments of this system. However, further refinements of the AMSS are needed (including computerization) before rolling out to full scale. Nevertheless, the AMSS has the potential to contribute to the empowerment of GPs, enabling them to better manage allergy patients in primary care.

## Abbreviations

AC service: asthma/COPD service
AMSS: allergy management support system
EAACI: European Academy of Allergy and Clinical Immunology
GPs: general practitioners
SD: standard deviation
sIgE: specific Immunoglobulin E
